# Supplementary material for: Deletion of the autism-related gene Chd8 alters activity-dependent transcriptional responses in mouse postmitotic neurons
Source: Commun Biol. 2023 Jun 2;6:593. doi: 10.1038/s42003-023-04968-y (PMC10238509; doi:10.1038/s42003-023-04968-y)
Supplement: Supplementary file 2 — Supplementary Information [file 42003_2023_4968_MOESM2_ESM.pdf]

Supplementary Information

Deletion of the autism-related gene *Chd8* alters activity-dependent transcriptional responses in mouse postmitotic neurons

Atsuki Kawamura & Masaaki Nishiyama

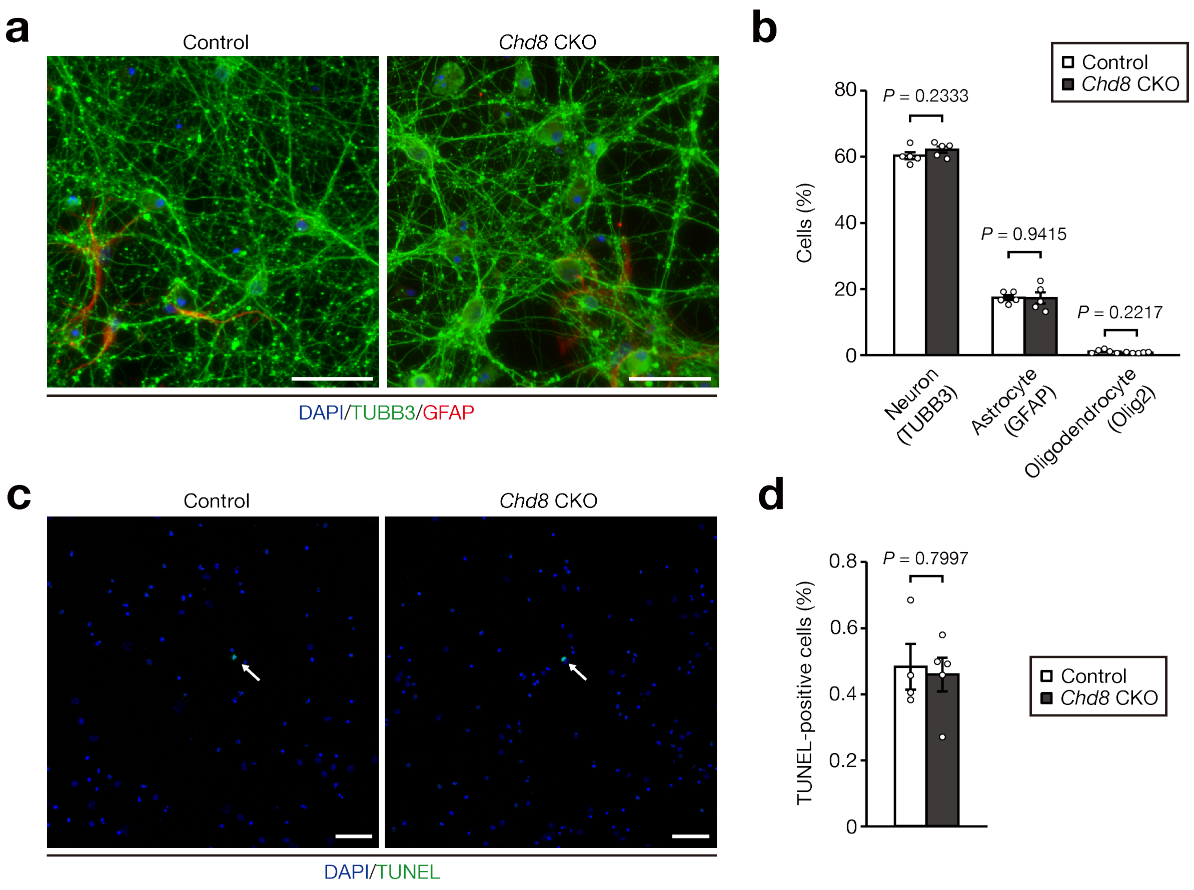

Supplementary Fig. 1 *Chd8* ablation does not affect the gross morphology or viability of primary neurons. a, b Immunofluorescence staining of TUBB3 (a marker for neurons) and GFAP (a marker for astrocytes) (**a**) and quantification of the number of TUBB3$^+$, GFAP$^+$, or Olig2$^+$ (a marker for oligodendrocytes) cells (**b**) for primary cultures prepared from the hippocampus of E18.5 *Chd8* CKO or control mice and maintained for 10 days (n = 5 mice per genotype). Nuclei were stained with 4′,6-diamidino-2-phenylindole (DAPI). Scale bars, 50 µm. **c, d** TUNEL staining (**c**) and quantification of the number of TUNEL-positive cells (**d**) for primary cultures prepared from the hippocampus of E18.5 *Chd8* CKO (n = 5) or control (n = 4) mice and maintained for 10 days. Nuclei were stained with DAPI. Scale bars, 50 µm. Arrows indicate TUNEL-positive cells. All quantitative data are means ± s.e.m. and were analyzed with the unpaired Student's t test.

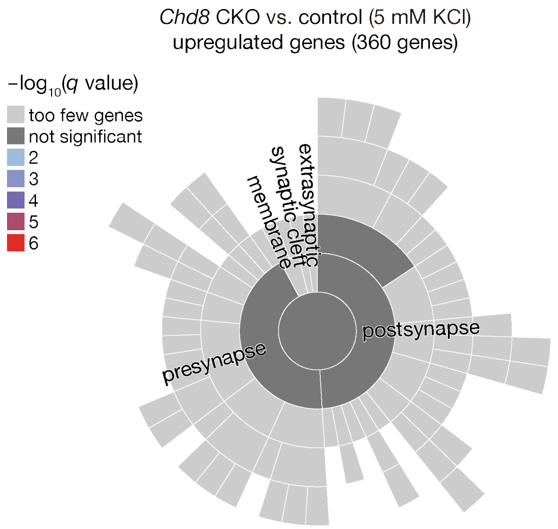

Supplementary Fig. 2 Upregulated genes in *Chd8* CKO neurons are not related to synapse function. SynGO analysis was performed for genes whose expression was upregulated (360 genes, FDR-adjusted *P* value of <0.05) in *Chd8* CKO neurons compared with control neurons under the 5 mM KCl condition as determined by RNA-seq analysis (*n* = 3 mice of each genotype, 1 male and 2 females for *Chd8* CKO mice and 2 males and 1 female for control mice). See Figure 1f.

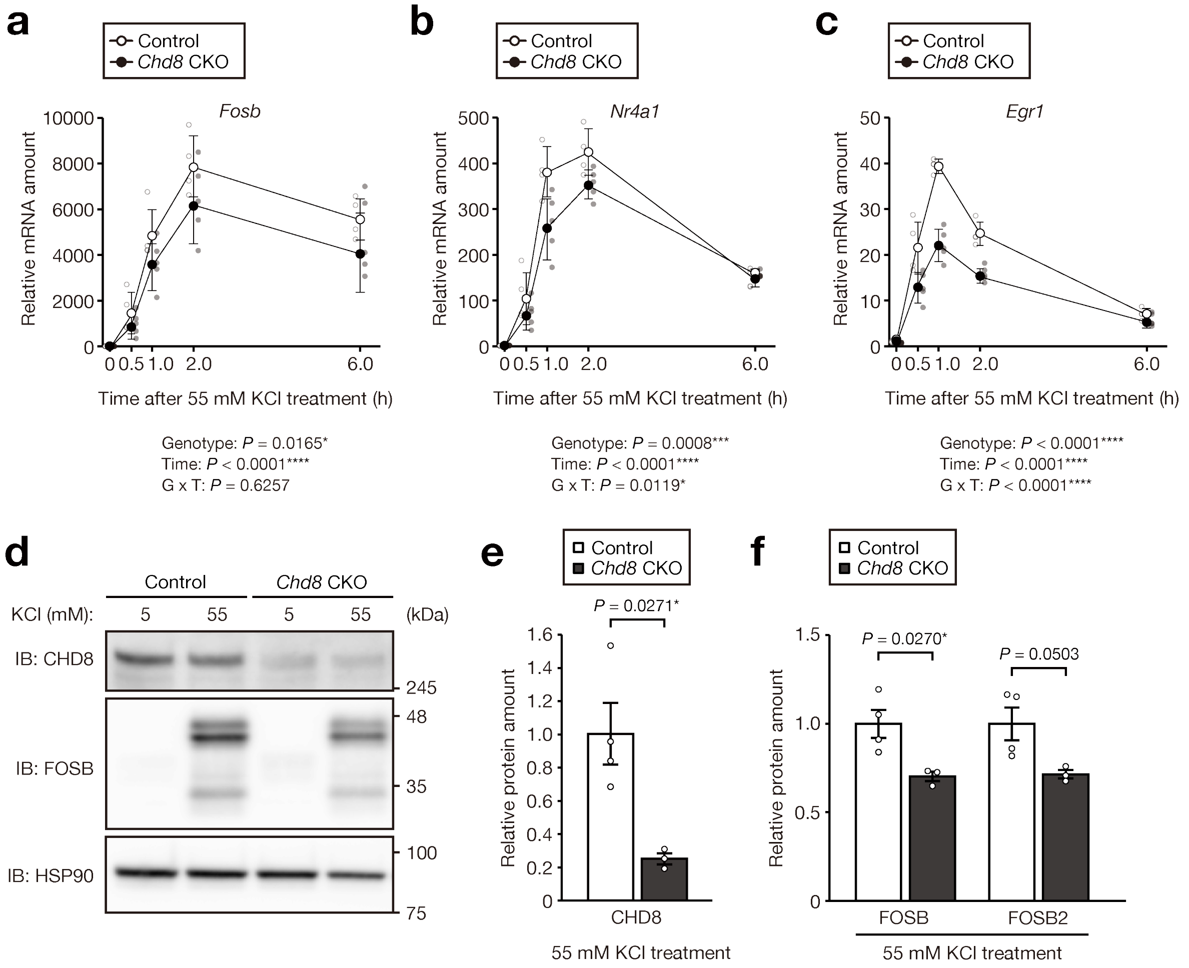

Supplementary Fig. 3 Expression of FOSB at the mRNA and protein levels is reduced in *Chd8* CKO neurons *in vitro*. **a–c** RT-qPCR analysis of the time course of mRNA abundance for *Fosb* (**a**), *Nr4a1* (**b**), and *Egr1* (**c**) in hippocampal neurons isolated from control (n = 4) or *Chd8* CKO (n = 5) mice and treated with 55 mM KCl for 0, 0.5, 1.0, 2.0, or 6.0 h. **d–f** Representative immunoblot (IB) analysis of CHD8, FOSB, and HSP90 (loading control) (**d**) and quantification of the abundance of CHD8 (**e**) and two isoforms of FOSB (**f**) for the primary neurons isolated from control (n = 4) or *Chd8* CKO (n = 3) mice and treated with 5 or 55 mM KCl for 2 h. All quantitative data are means ± s.e.m. *$P < 0.05$, ***$P < 0.001$, ****$P < 0.0001$ (two-way repeated ANOVA (**a**–**c**) or unpaired Student's t test (**e**, **f**)). Uncropped blots are provided in Supplementary Figure 8.

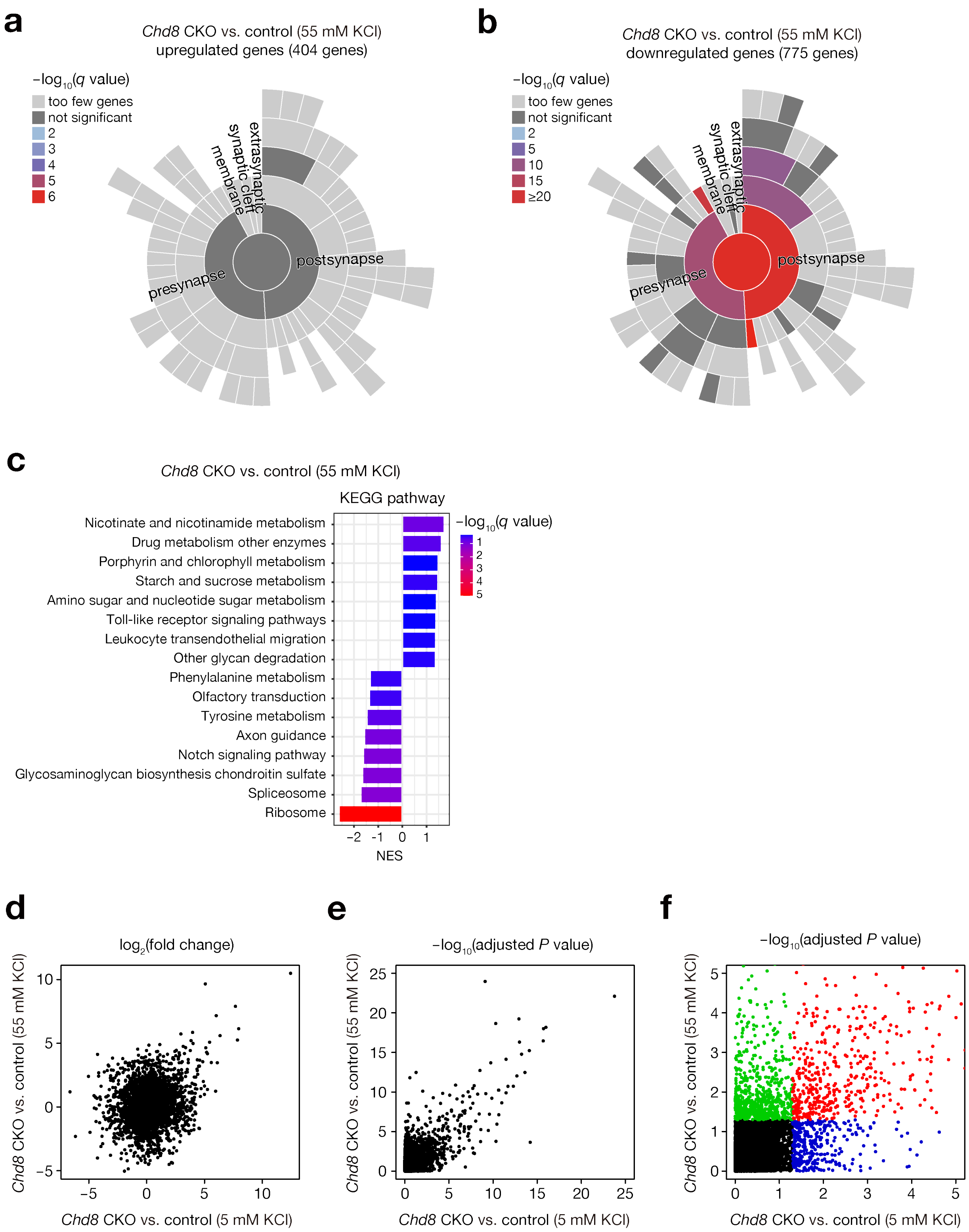
a
Chd8 CKO vs. control (55 mM KCl)
upregulated genes (404 genes)
−log10(q value)
too few genes
not significant
2
3
4
5
6
extrasynaptic
synaptic cleft
membrane
postsynapse
presynapse
b
Chd8 CKO vs. control (55 mM KCl)
downregulated genes (775 genes)
−log10(q value)
too few genes
not significant
2
5
10
15
≥20
c
Chd8 CKO vs. control (55 mM KCl)
KEGG pathway
Nicotinate and nicotinamide metabolism
Drug metabolism other enzymes
Porphyrin and chlorophyll metabolism
Starch and sucrose metabolism
Amino sugar and nucleotide sugar metabolism
Toll-like receptor signaling pathways
Leukocyte transendothelial migration
Other glycan degradation
Phenylalanine metabolism
Olfactory transduction
Tyrosine metabolism
Axon guidance
Notch signaling pathway
Glycosaminoglycan biosynthesis chondroitin sulfate
Spliceosome
Ribosome
NES
d
log2(fold change)
Chd8 CKO vs. control (55 mM KCl)
Chd8 CKO vs. control (5 mM KCl)
e
−log10(adjusted P value)
Chd8 CKO vs. control (55 mM KCl)
Chd8 CKO vs. control (5 mM KCl)
f
−log10(adjusted P value)
Chd8 CKO vs. control (55 mM KCl)
Chd8 CKO vs. control (5 mM KCl)

Supplementary Fig. 4 Translation- and neuron-related gene expression is downregulated by *Chd8* ablation in postmitotic neurons under the 55 mM KCl condition. a, b SynGO analysis of genes whose expression was upregulated (404 genes) (**a**) or downregulated (775 genes) (**b**) in *Chd8* CKO neurons compared with control neurons under the 55 mM KCl condition. **c** GSEA for KEGG pathways in *Chd8* CKO neurons compared with control neurons under the 55 mM KCl condition. NES, normalized enrichment score. **d–f** Scatter plots for $\log_2$(fold change) (**d**) and $-\log_{10}$(FDR-adjusted *P* value) (**e**) for *Chd8* CKO neurons compared with control neurons under the 5 mM KCl condition versus those under the 55 mM KCl condition (see also Supplementary Data 4). A scatter plot expanded between $-\log_{10}$(FDR-adjusted *P* value) of 0 and 5 is shown in **f**. Differentially expressed genes (FDR-adjusted *P* value of <0.05) under both 5 and 55 mM KCl conditions are highlighted in red, those under only the 5 mM KCl condition are highlighted in blue, and those under only the 55 mM KCl condition are highlighted in green.

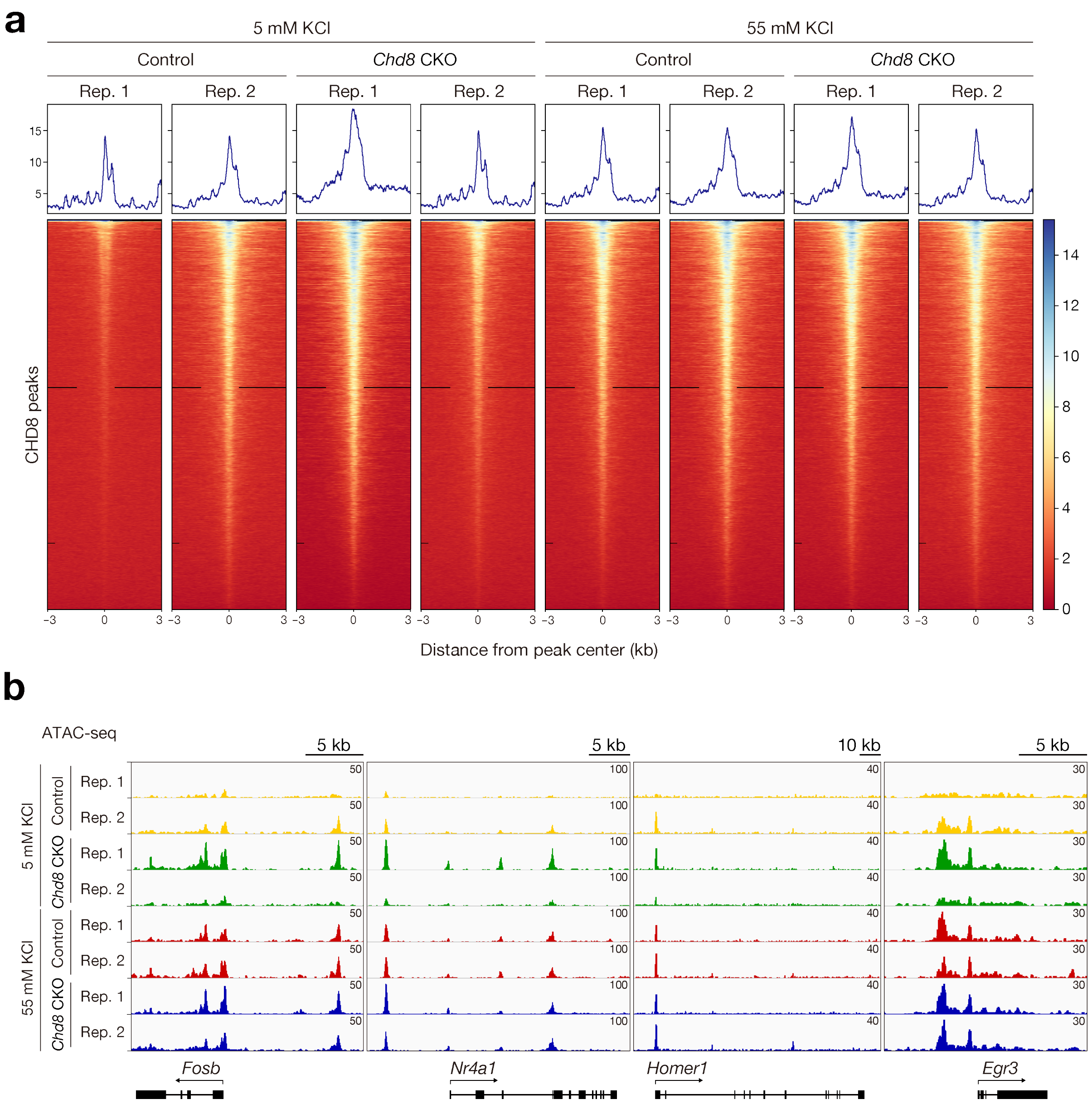

Supplementary Fig. 5 Replication of ATAC-seq analysis for postmitotic neurons under the 5 or 55 mM KCl condition. a Grouping of signal density and heat maps of ATAC-seq peaks in the region spanning 3 kb upstream to 3 kb downstream of the center of CHD8 binding peaks (n = 2 mice per condition). Rep., replicate. **b** ATAC-seq signals of representative activity-dependent genes viewed in the Integrative Genomics Viewer browser for control and *Chd8* CKO neurons treated with 5 or 55 mM KCl. See Figure 3b, c.

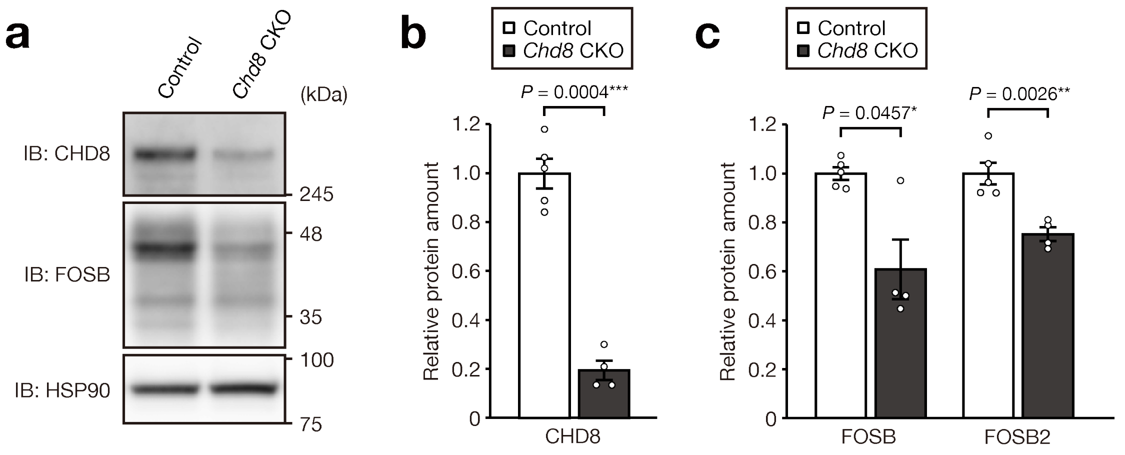

Supplementary Fig. 6 Expression of FOSB at the mRNA and protein levels is reduced in *Chd8* CKO mouse neurons *in vivo*. Representative immunoblot (IB) analysis of CHD8, FOSB, and HSP90 (loading control) (**a**) and quantification of the abundance of CHD8 (**b**) and two isoforms of FOSB (**c**) are shown for the hippocampus of control (n = 5) and *Chd8* CKO (n = 4) mice treated with KA for 60 min. Quantitative data are means ± s.e.m. *P < 0.05, **P < 0.01, ***P < 0.001 (unpaired Student's t test). Uncropped blots are provided in Supplementary Figure 8.

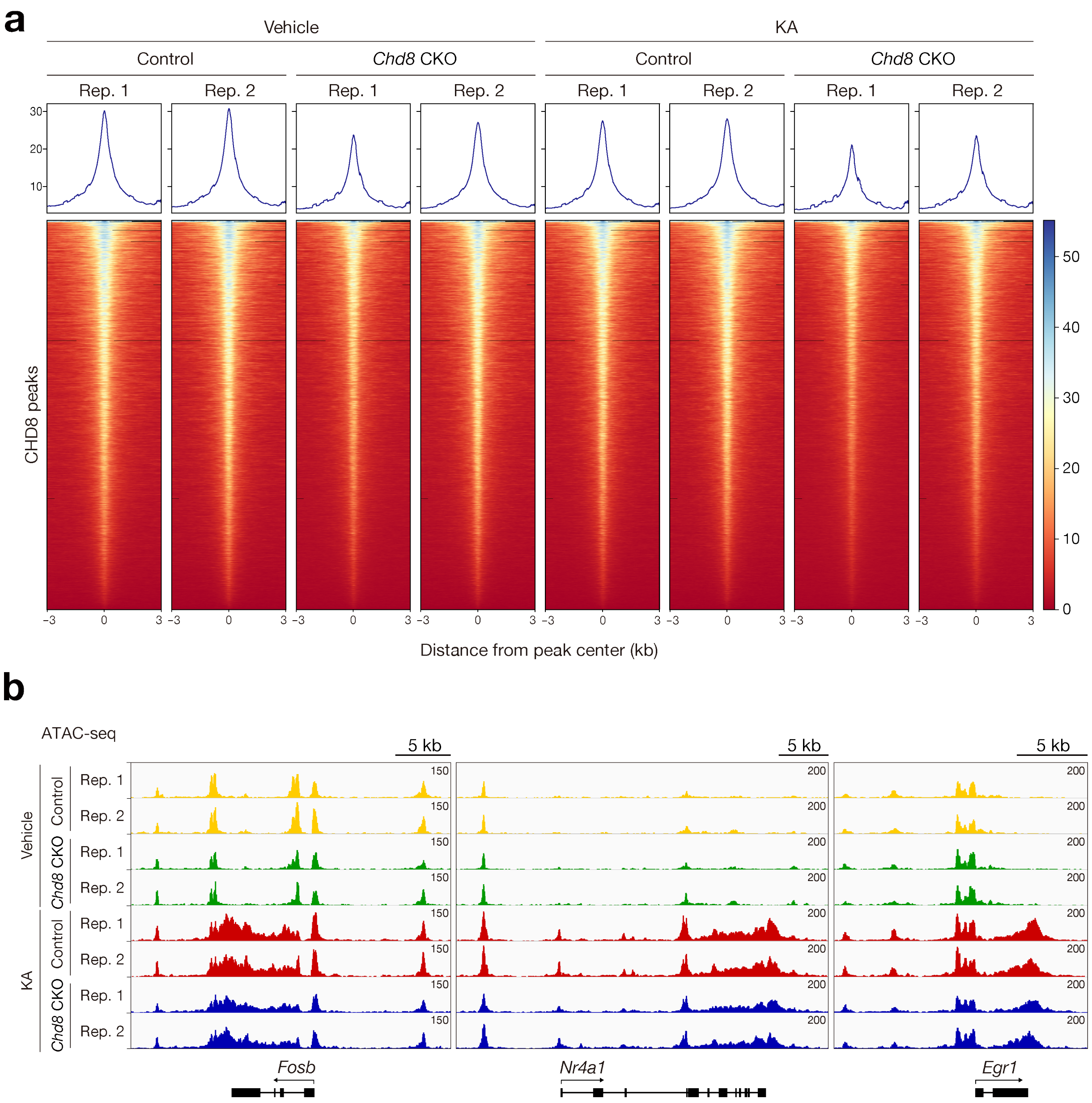

Supplementary Fig. 7 Replication of ATAC-seq analysis for the hippocampus after KA treatment. **a** Grouping of signal density and heat maps of ATAC-seq peaks in the region spanning 3 kb upstream to 3 kb downstream of the center of CHD8 binding peaks (n = 2 mice per condition: 1 female (replicate (Rep.) 1) and 1 male (Rep. 2) for *Chd8* CKO and control mice treated with KA, 2 males (Rep. 1 and 2) for *Chd8* CKO and control mice treated with vehicle). **b**

ATAC-seq signals of representative activity-dependent genes viewed in the Integrative Genomics Viewer browser for the hippocampus of *Chd8* CKO and control mice as in **a**. See Figure 4e, f.

Supplementary Figure 3d

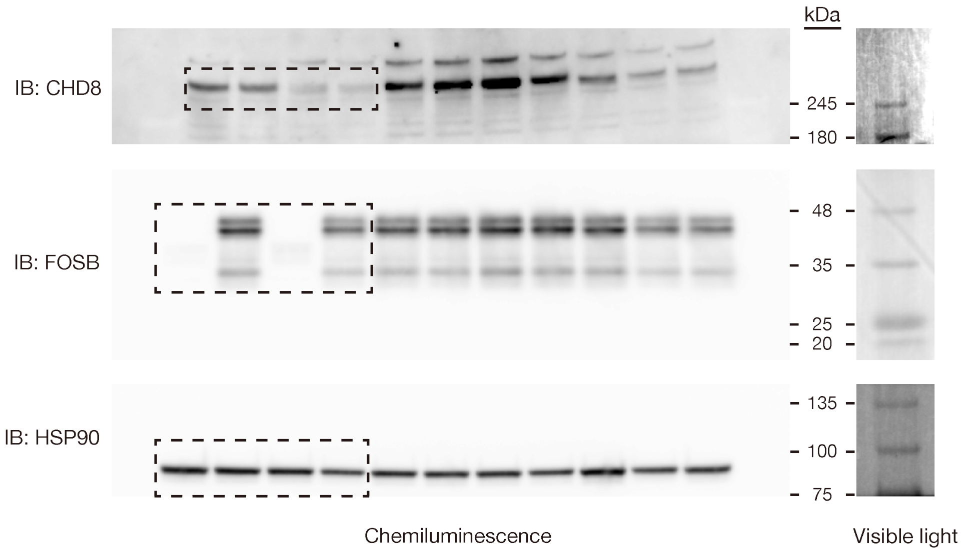

Supplementary Figure 6a

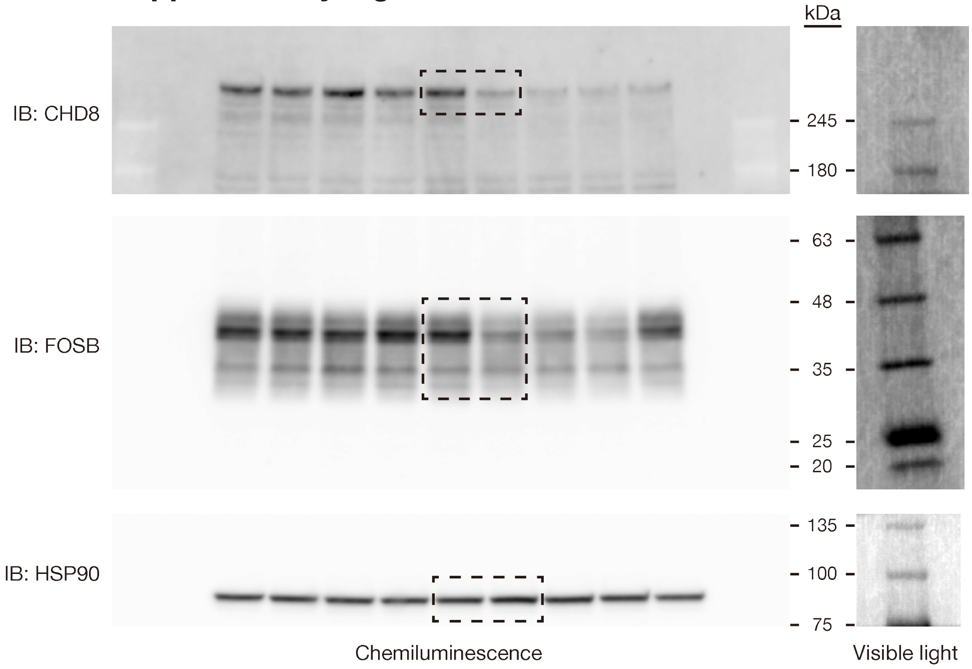

Supplementary Fig. 8 Uncropped immunoblots for data shown in Supplementary Figures 3d and 6a.
