# Supplementary material for: Deletion of the autism-related gene Chd8 alters activity-dependent transcriptional responses in mouse postmitotic neurons
Source: Commun Biol. 2023 Jun 2;6:593. doi: 10.1038/s42003-023-04968-y (PMC10238509; doi:10.1038/s42003-023-04968-y)
Supplement: Supplementary file 3 — Description of Additional Supplementary Files [file 42003_2023_4968_MOESM3_ESM.docx]

**Description of Additional Supplementary Files**

**File name:** Supplementary Data 1

**File Description:** This file contains results (fold change and *P* value) for total genes of RNA-seq analysis performed in this study. Supplementary Table 1–4.

**File name:** Supplementary Data 2

**File Description:** This file contains the numerical source data for all graphs and charts.
