# Supplementary material for: Deletion of the autism-related gene Chd8 alters activity-dependent transcriptional responses in mouse postmitotic neurons
Source: Commun Biol. 2023 Jun 2;6:593. doi: 10.1038/s42003-023-04968-y (PMC10238509; doi:10.1038/s42003-023-04968-y)
Supplement: Supplementary file 6 — nr-reporting-summary [file 42003_2023_4968_MOESM6_ESM.pdf]

nature portfolio

Corresponding author(s): Masaaki Nishiyama

Last updated by author(s): May 16, 2023

Reporting Summary

Nature Portfolio wishes to improve the reproducibility of the work that we publish. This form provides structure for consistency and transparency in reporting. For further information on Nature Portfolio policies, see our Editorial Policies and the Editorial Policy Checklist.

Statistics

For all statistical analyses, confirm that the following items are present in the figure legend, table legend, main text, or Methods section.

n/a	Confirmed	
☐	☒	The exact sample size (*n*) for each experimental group/condition, given as a discrete number and unit of measurement
☐	☒	A statement on whether measurements were taken from distinct samples or whether the same sample was measured repeatedly
☐	☒	The statistical test(s) used AND whether they are one- or two-sided<br>*Only common tests should be described solely by name; describe more complex techniques in the Methods section.*
☐	☒	A description of all covariates tested
☐	☒	A description of any assumptions or corrections, such as tests of normality and adjustment for multiple comparisons
☐	☒	A full description of the statistical parameters including central tendency (e.g. means) or other basic estimates (e.g. regression coefficient) AND variation (e.g. standard deviation) or associated estimates of uncertainty (e.g. confidence intervals)
☐	☒	For null hypothesis testing, the test statistic (e.g. *F*, *t*, *r*) with confidence intervals, effect sizes, degrees of freedom and *P* value noted<br>*Give P values as exact values whenever suitable.*
☒	☐	For Bayesian analysis, information on the choice of priors and Markov chain Monte Carlo settings
☒	☐	For hierarchical and complex designs, identification of the appropriate level for tests and full reporting of outcomes
☒	☐	Estimates of effect sizes (e.g. Cohen's *d*, Pearson's *r*), indicating how they were calculated

Our web collection on statistics for biologists contains articles on many of the points above.

Software and code

Policy information about availability of computer code

Data collection	The reads obtained from RNA-seq experiments were uniquely mapped to the mouse (mm10) genome with the use of HISAT2 (version 2.1.0), and counted with featureCounts (version 2.0.0). The reads obtained from ATAC-seq experiments in this study and ChIP-seq data from a previous study were uniquely mapped to the mouse (mm10) genome with the use of Bowtie software (version 2.2.3), and duplicated reads were removed with samtools (version 1.9).
Data analysis	RNA-seq data were analyzed by using DESeq2 (version 1.26.0) and GSEA software (version 4.2.1) in R software. ATAC-seq and ChIP-seq data were analyzed by using MACS peak caller (version 2.1.1) and deepTools (version 3.5.0).

For manuscripts utilizing custom algorithms or software that are central to the research but not yet described in published literature, software must be made available to editors and reviewers. We strongly encourage code deposition in a community repository (e.g. GitHub). See the Nature Portfolio guidelines for submitting code & software for further information.

Data

Policy information about availability of data

All manuscripts must include a data availability statement. This statement should provide the following information, where applicable:

- Accession codes, unique identifiers, or web links for publicly available datasets
- A description of any restrictions on data availability
- For clinical datasets or third party data, please ensure that the statement adheres to our policy

The accession numbers of the RNA-seq and ATAC-seq data reported in this study are DRA014131, DRA016165, and DRA016198. The ChIP-seq data from the previous study analyzed in Fig. 3 is available on DRA003116.

Research involving human participants, their data, or biological material

Policy information about studies with human participants or human data. See also policy information about sex, gender (identity/presentation), and sexual orientation and race, ethnicity and racism.

Reporting on sex and gender	NA
Reporting on race, ethnicity, or other socially relevant groupings	NA
Population characteristics	NA
Recruitment	NA
Ethics oversight	NA

Note that full information on the approval of the study protocol must also be provided in the manuscript.

Field-specific reporting

Please select the one below that is the best fit for your research. If you are not sure, read the appropriate sections before making your selection.

☒ Life sciences ☐ Behavioural & social sciences ☐ Ecological, evolutionary & environmental sciences

For a reference copy of the document with all sections, see nature.com/documents/nr-reporting-summary-flat.pdf

Life sciences study design

All studies must disclose on these points even when the disclosure is negative.

Sample size	No statistical methods were used to predetermine sample sizes, but sample size was similar to those generally employed in the field.
Data exclusions	No data were excluded from the analyses.
Replication	The experimental findings were reliably reproduced through repeated experiments.
Randomization	All individual mice or samples were randomly allocated to the experimental groups.
Blinding	The experimenters were blinded to mouse genotype during data collection.

Reporting for specific materials, systems and methods

We require information from authors about some types of materials, experimental systems and methods used in many studies. Here, indicate whether each material, system or method listed is relevant to your study. If you are not sure if a list item applies to your research, read the appropriate section before selecting a response.

Materials & experimental systems

n/a	Involved in the study
☐	☒ Antibodies
☒	☐ Eukaryotic cell lines
☒	☐ Palaeontology and archaeology
☐	☒ Animals and other organisms
☒	☐ Clinical data
☒	☐ Dual use research of concern
☒	☐ Plants

Methods

n/a	Involved in the study
☒	☐ ChIP-seq
☒	☐ Flow cytometry
☒	☐ MRI-based neuroimaging

Antibodies

Antibodies used

Rabbit polyclonal antibodies to CHD8 were generated in-house and used for immunoblot analysis. Other antibodies included those to TUBB3 (ab78078, Abcam), to GFAP (IR524, DAKO), and to Olig2 (AB9610, Millipore) for immunofluorescence staining, those to FOSB (ab184938, Abcam) and to HSP90 (610419, BD Biosciences) for immunoblot analysis, and those to H3K4me3 (ab8580, Abcam) and to RNA polymerase II (91151, Active Motif) for ChIP.

Validation

Antibody validation was reported from the companies that provide the antibodies or from cited literatures.

Animals and other research organisms

Policy information about studies involving animals; ARRIVE guidelines recommended for reporting animal research, and Sex and Gender in Research

Laboratory animals

Cre recombinase–mediated Chd8 knockout (CAG-CreER/Chd8F/F) and control (Chd8F/F) mice were used in this study. CAG-CreER/Chd8F/F mice were backcrossed onto the C57BL/6J line for at least nine generations. Primary neurons were isolated from the hippocampus of mice at E18.5. Behavioral tests were performed with male mice at 11 to 18 weeks of age.

Wild animals

NA

Reporting on sex

Primary neuron culture was performed without distinguishing the sex of the mouse. Male and female mice at 11 to 18 weeks of age was used for behavioral analysis.

Field-collected samples

NA

Ethics oversight

All mouse experiments were approved by the Animal Ethics Committee of Kanazawa University.

Note that full information on the approval of the study protocol must also be provided in the manuscript.
